# A Vacuolar Processing Enzyme *RsVPE1* Gene of Radish Is Involved in Floral Bud Abortion under Heat Stress

**DOI:** 10.3390/ijms140713346

**Published:** 2013-06-27

**Authors:** Jing Zhang, Qing-Fei Li, Wei-Wei Huang, Xiao-Yong Xu, Xin-Ling Zhang, Mai-Xia Hui, Ming-Ke Zhang, Lu-Gang Zhang

**Affiliations:** 1 College of Horticulture, Northwest A&F University, Yangling 712100, Shaanxi, China; E-Mails: 111zhangjing@163.com (J.Z.); chenchao_20061225@126.com (Q.-F.L.); xuxy7926@163.com (X.-Y.X.); zxl99988@126.com (X.-L.Z.); maixiahui@163.com (M.-X.H.); zhangmk1101@nwsuaf.edu.cn (M.-K.Z.); 2 State Key Laboratory of Crop Stress Biology in Arid Areas, Northwest A&F University, Yangling 712100, Shaanxi, China; E-Mail: huangwei-0210@nwsuaf.edu.cn; 3 College of Life Sciences, Northwest A&F University, Yangling 712100, Shaanxi, China

**Keywords:** radish, vacuolar processing enzyme, floral bud abortion, heat stress

## Abstract

Radish floral bud abortion (FBA) is an adverse biological phenomenon that occurs during reproduction. Although FBA is a frequent occurrence, its molecular mechanism remains unknown. A transcript-derived fragment (TDF72), which was obtained by cDNA amplified fragment length polymorphism (cDNA-AFLP), was up-regulated in the aborted buds and exhibited 89% sequence homology with the *AtγVPE* gene. In this study, TDF72 was used to clarify the role of VPE in FBA by isolation of the VPE gene *RsVPE1* from radish flower buds. The full-length genomic DNA was 2346 bp including nine exons and eight introns. The full-length cDNA was 1825 bp, containing a complete open reading frame (ORF) of 1470 bp, which encoded a predicted protein containing 489 amino acid residues, with a calculated molecular mass of 53.735 kDa. Expression analysis demonstrated that *RsVPE1* was expressed in all tested organs of radish at different levels. Highest expression was detected in aborted flower buds, suggesting that *RsVPE1* has a role in FBA. In order to analyze the role of *RsVPE1* in FBA, *RsVPE1* was overexpressed in transgenic *Arabidopsis thaliana* plants. Aborted flower buds appeared in transgenic plants subjected to heat stress. In addition, *RsVPE1* expression in the transgenic plants reached a maximum when subjected to heat stress for 24 h and increased by 2.1-fold to 2.8-fold in three homozygous transgenic lines. These results indicated that *RsVPE1* led to FBA when its expression levels exceeded a particular threshold, and provided evidence for the involvement of *RsVPE1* in promoting FBA under heat stress.

## 1. Introduction

**Figure 1:**
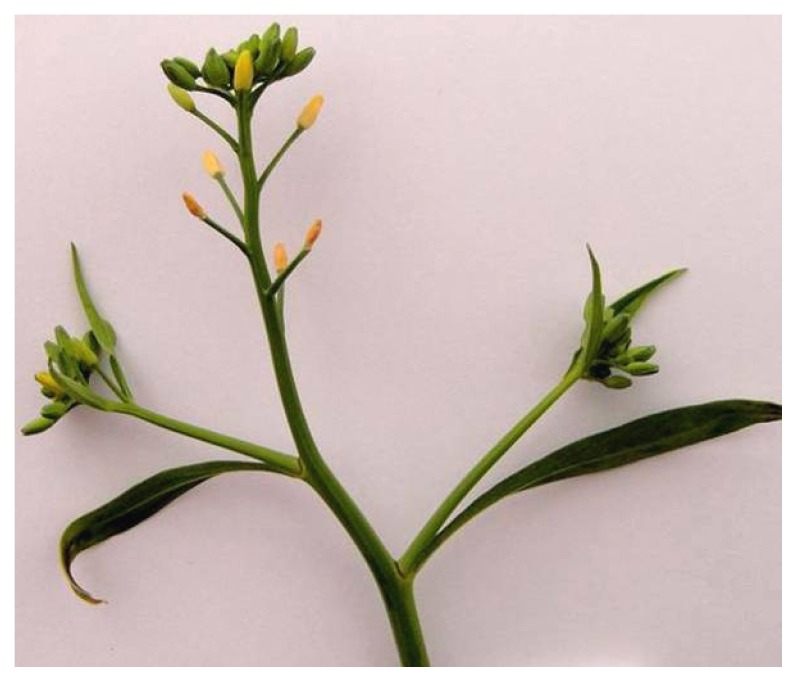
Floral bud abortion (FBA) of radish.

Floral bud abortion (FBA) is an adverse biological phenomenon affecting radish (*Raphalus sativus* L.) reproduction, which is commonly associated with radish breeding practices and the creation of radish germplasm resources. During FBA, the floral bud first stops growing and then gradually turns from green to yellow, until it finally dries out completely (Figure 1). Plants exhibiting FBA are often eliminated during artificial breeding practices or lost naturally, even if they have good economic characteristics or specific traits, which is ultimately harmful for the selection of new radish cultivars and conservation of biodiversity. FBA is a widespread phenomenon in plant reproduction and evolution, having been observed in *Cucurbita texana* [1], dwarf bean [2], cowpea [3], rape [4], Chinese cabbage [5], radish [6,7], and the model plant *Arabidopsis thaliana* [8]. FBA in plants, including *C. texana*, cowpea, dwarf beans and *A. thaliana*, is most commonly thought to result from abiotic stress. Despite the widespread occurrence of FBA, there is limited knowledge about its molecular mechanism, with only a few preliminary studies published to date. Zhang *et al.* analyzed gene expression during 10 continuous developmental periods of FBA in Chinese cabbage using cDNA-AFLP, and found 72 novel expressed sequence tags (ESTs) related to the aborted buds [5]. Jia *et al.* evaluated the aborted and normal buds of radish and obtained 107 differentially expressed fragments related to FBA. During FBA in radish, characteristics of programmed cell death (PCD) were detected at the DNA level [6,7].

Numerous reports indicate that proteases with caspase-like activity exist in plants and mediate processes of cell death during development and stress responses [9,10]. Previous work has described the vacuole-localized cysteine proteases called vacuolar processing enzymes (VPEs), which were originally discovered in the maturation of seed storage proteins [11]. Phylogenic, as well as expression and sequence analysis of VPEs have indicated that they are classified into the three following categories: seed type, seed coat type, and vegetative type [12,13]. Two vegetative type VPE proteins have been identified in *Arabidopsis*, namely *αVPE* and *γVPE*, both of which are mainly expressed in vegetative tissues. The mRNA expression of these genes is up-regulated in response to various abiotic stresses, such as wounding, senescence, and treatment with hormones including jasmonic acids, ethylene, and salicylic acid [14–16]. Furthermore, it was reported that a functional VPE protein acts as a critical factor initiating hypersensitive cell death in response to infection with tobacco mosaic virus (TMV) and *Pseudomonas syringae* pv. *phaseolicola* in tobacco [17]. Studies have provided evidence that *AtγVPE* exhibiting a caspase-1 activity is a key molecule in toxin-induced cell death [18]. TDF72, a transcript-derived fragment obtained by cDNA-AFLP in our previous study, was up-regulated in the aborted buds and exhibited 89% sequence homology with the *AtγVPE* gene, suggesting that VPE has a role in FBA, since *VPE* has been known to link with PCD. Previous research into plant VPEs has focused mainly on plant senescence and pathogen-induced hypersensitive cell death, but its role in FBA is poorly understood.

In this study, TDF72 was used to analyze the role of VPE in FBA, by isolation of the VPE gene *RsVPE1* from radish flower buds. Expression of the *RsVPE1* gene was analyzed in different tissues of radish plants. In addition, to examine the effect increased *RsVPE1* expression on FBA, *RsVPE1* was overexpressed under the CaMV 35S promoter in transgenic *Arabidopsis* thaliana plants.

## 2. Results and Discussion

### 2.1. Molecular Cloning and Sequence Analysis of *RsVPE1*

**Figure 2:**
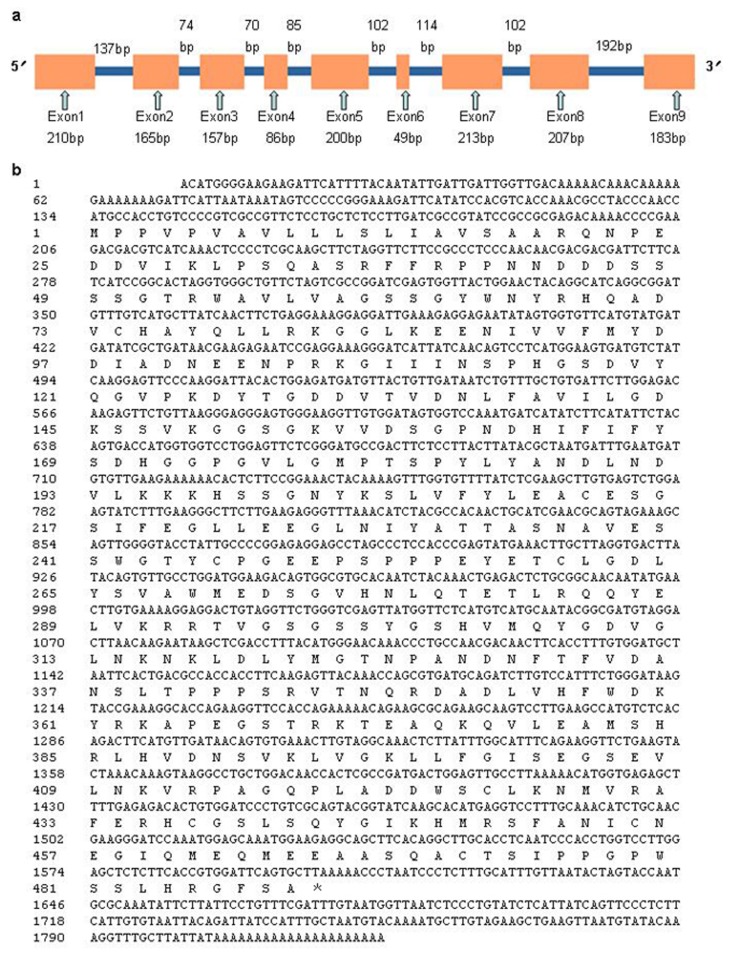
Schematic diagram and nucleotide sequence of *RsVPE1*. (**a**) Schematic diagram of the genomic *RsVPE1* sequence. Exons are indicated by hollow arrows followed by their names and sizes. Orange and blue boxes indicate the exons and introns, respectively; (**b**) Schematic diagram of the full-length *RsVPE1* cDNA sequence. The numbers on the left are the nucleotide and amino acid positions.

To investigate the role of VPEs in FBA, the *RsVPE1* gene was identified and characterized. The complete 5′ and 3′ regions of the putative *RsVPE1* gene were isolated by rapid amplification of cDNA ends (RACE) from radish flower buds. Based on the sequence of TDF72, the 3′-cDNA end and 5′-cDNA end were determined by RACE to be 937 bp and 1030 bp in length, respectively. The full-length cDNA of the *RsVPE1* was 1825 bp, containing the start codon ATG, the stop codon TAG, a 5′-untranslated region (UTR) of 133 bp, a complete ORF of 1470 bp and a 3′-UTR of 222 bp with a poly(A) tail of 21 bp (Figure 2b). The long ORF encoded a predicted protein containing 489 amino acid residues, with a calculated molecular mass of 53.735 kDa, and an isoelectric point of 5.38. The full-length gDNA of the *RsVPE1* gene was 2346 bp including nine exons and eight introns (Figure 2a). InterProScan showed conserved domain features of Peptidase_C13 in the predicted RsVPE1 protein, which had sequence and functional similarity with plant VPEs.

**Figure 3:**
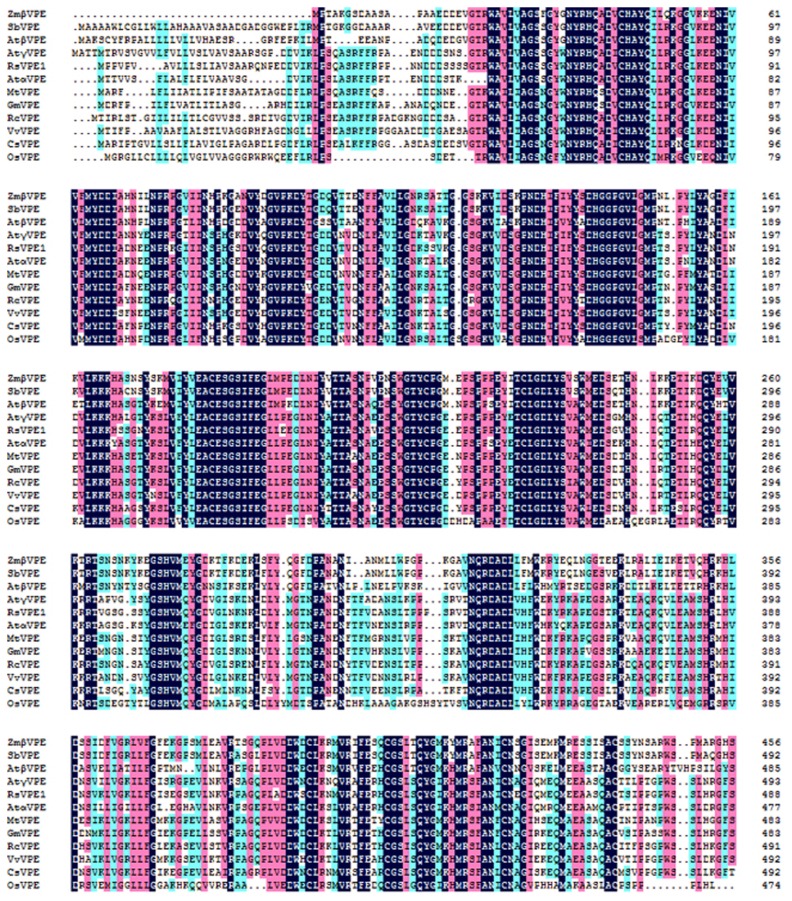
Comparison of the predicted protein sequence of RsVPE1 with other vacuolar processing enzyme (VPE) proteins. The accession numbers of these proteins in the GenBank database are as follows: AtαVPE (NP_180165.1), AtβVPE (NP_176458.1), AtγVPE (NP_195020.1), CsVPE (XP_004142919.1), GmVPE (XP_003525979.1), MtVPE (XP_003603121.1), OsVPE (NP_0010565054.1), RcVPE (XP_002516472.1), SbVPE (XP_002448237.1), VvVPE (XP_002276759.1), ZmβVPE (ACG47915). At, *Arabidopsis thaliana*; Cs, *Cucumis sativus*; Gm, *Glycine max*; Mt, *Medicago truncatula*; Os, *Oryza sativa*; Rc, *Ricinus communis*; Rs, *Raphalus sativus*; Sb, *sorghum bicolor*; Vv, *Vitis vinifera*; Zm, *Zea mays*. Black indicates identical amino acids among the different species. Pink and blue represent similar amino acids.

Multiple amino acid sequence alignment revealed that the predicted amino acid sequence of RsVPE1 displayed homology with other proteins (Figure 3), including *Arabidopsis thaliana* gamma-VPE (NP_195020.1, 90%), *Arabidopsis thaliana* alpha-VPE (NP_180165.1, 80%), *Vitis vinifera* VPE (XP_002276759.1, 77%), *Ricinus communis* VPE (XP_002516472.1, 77%), *Glycine max* VPE (XP_003525979.1, 75%), *Medicago truncatula* VPE (XP_003603121.1, 74%), *Cucumis sativus* VPE (XP_004142919.1,72%), *Sorghum bicolor* VPE (XP_002448237.1, 63%), *Arabidopsis thaliana* beta-VPE(NP_176458.1, 63%); *Zea mays* beta-VPE (ACG47915, 62%) and *Oryza sativa* VPE (NP_0010565054.1, 59%).

**Figure 4:**
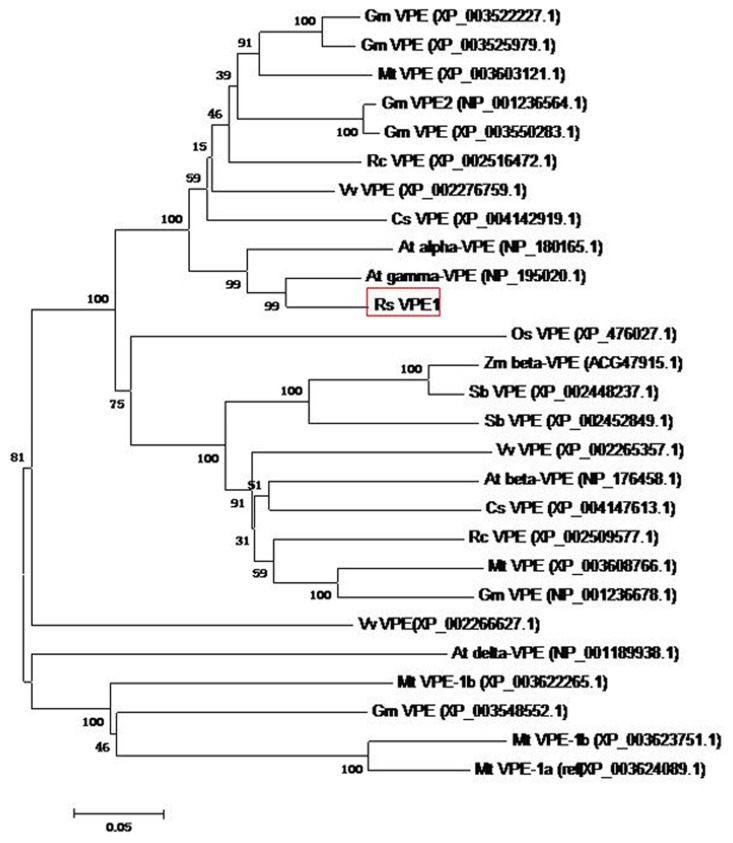
Phylogenic tree of VPE proteins. The rooted gene tree (majority-rule consensus from 1000 bootstrap replicates) resulted from heuristic searching in Mega5.0. Bootstrap values are indicated at each branch node. At, *Arabidopsis thaliana*; Cs, *Cucumis sativus*; Gm, *Glycine max*; Mt, *Medicago truncatula*; Os, *Oryza sativa*; Rc, *Ricinus communis*; Rs, *Raphalus sativus*; Sb, *sorghum bicolor*; Vv, *Vitis vinifera*; Zm, *Zea mays*. Bootstrap values are indicated at each branch node. *RsVPE1* was marked with red box. GenBank accession numbers are in parentheses after each species and protein name. Scale bar indicates similarity coefficient.

Subsequently, to evaluate the molecular evolutionary relationships of RsVPE1 against other VPEs, a phylogenic tree was constructed. RsVPE1 exhibited the closest relationship with AtγVPE (Figure 4), belonging to a novel group of cysteine proteinases and is up-regulated in association with various types of cell death and under stress conditions [12,14–17]. Although the result showed that the *RsVPE1* gene shared 90% identity with *AtγVPE*, there are differences in the predicted protein sequences of these two genes. The predicted protein of RsVPE1 is made of 489 amino acid residues. However, the predicted protein of AtγVPE is consisted of 494 amino acid residues. Such differences would be reflected in the functions of these vegetative type *VPEs*.

### 2.2. Analysis of Tissue-Specific *RsVPE1* Expression in Radish Plants

**Figure 5:**
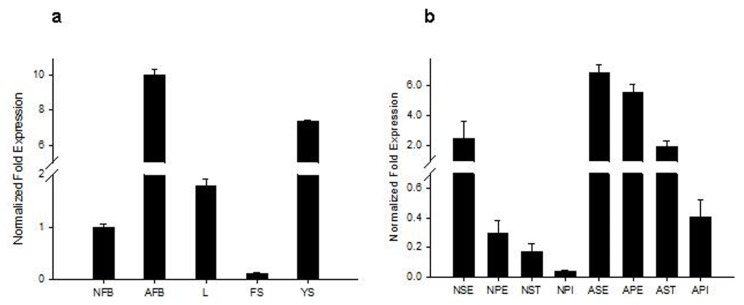
Expression analysis of *RsVPE1* gene in radish plants. *RsVPE1* expression in (**a**) radish plants and (**b**) the four parts of radish flower buds. NFB, normal flower buds; AFB, aborted flower buds; L, leaves; FS, flower stalks; YS, young siliques; NSE, sepals of normal flower buds; NPE, petals of normal flower buds; NST, stamens of normal flower buds; NPI, pistils of normal flower buds; ASE, sepals of aborted flower buds; APE, petals of aborted flower buds; AST, stamens of aborted flower buds; API, pistils of aborted flower buds. Error bars represent the mean ± SD of three independent biological replicates.

Real-time PCR was performed to determine the tissue-specific expression of *RsVPE1* in radish plants. *RsVPE1* transcripts were produced in all the analyzed radish tissues including leaves, flower stalks, flower buds (normal and aborted) and young siliques (Figure 5a). However, the expression levels of *RsVPE1* gene varied in different tissues. The highest *RsVPE1* expression was detected in aborted flower buds, which indicates that *RsVPE1* plays an important role in FBA. Furthermore, the *RsVPE1* gene expression in different organs of radish flower buds were analyzed (Figure 5b). Interestingly, the levels of *RsVPE1* transcripts were higher in the different organs of aborted flower buds than in the corresponding organs of normal flower buds, with the highest expression levels in the sepals and the lowest expression levels in the pistils. It has been reported that the cells of the sepal, petal and anther in aborted flower buds exhibited obvious degeneration, resulting in abnormality of flower bud development. However, degeneration of pistil tissue was not observed in aborted flower buds [7]. In this study, *RsVPE1* expression levels in the pistils were the lowest among the four parts of flower bud, suggesting that *RsVPE1* almost does not affect the development of pistils.

### 2.3. RsVPE1-Overexpressing in Transgenic *Arabidopsis* Plants

To further investigate the role of *RsVPE1* in plants, we generated transgenic *Arabidopsis* expressing the *RsVPE1* cDNA under the control of the CaMV 35S promoter, and eight homozygous transgenic lines were obtained by antibiotic resistance screening and PCR confirmation. Three independent homozygous transgenic lines (TL3, TL78 and TL83) were randomly selected for further analysis. The expression level was checked by real-time PCR analysis. To examine the possible phenotype of homozygous transgenic lines, T_3_ progeny of the *RsVPE1*-overexpressed lines and the Columbia ecotype (Col-0) plants were grown in the greenhouse under normal conditions. Compared with Col-0 plants, no obvious morphological or developmental abnormalities were observed in transgenic plants when they were grown in artificial soil at normal temperatures in the greenhouse.

### 2.4. FBA in RsVPE1-Overexpressing Transgenic *Arabidopsis* Plants under Heat Stress

**Figure 6:**
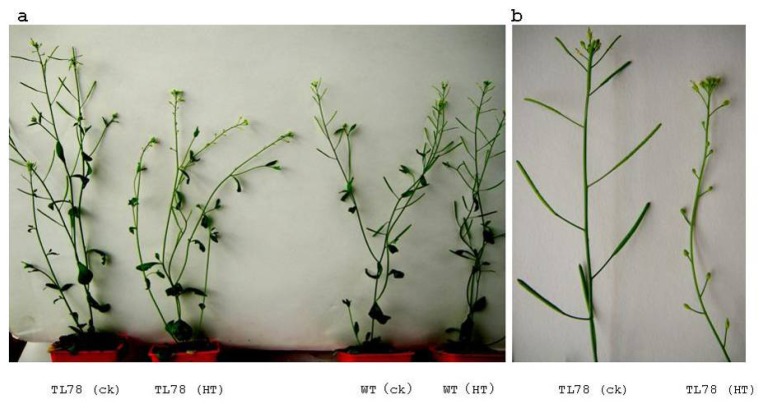
FBA in transgenic *Arabidopsis* under heat stress. (**a**) The image of homozygous transgenic *Arabidopsis* line and wild-type plant on the eighth day after heat stress; (**b**) Local amplification of (**a**). TL78(ck), transgenic plants at normal temperature; TL78(HT), transgenic plant on the eighth day after treated with 32 ± 0.5 °C for 36 h; WT(ck), Col-0 of *Arabidopsis thaliana* at normal temperature; WT(HT), Col-0 of *Arabidopsis thaliana* on the eighth day after treated with 32 ± 0.5 °C for 36 h.

**Figure 7:**
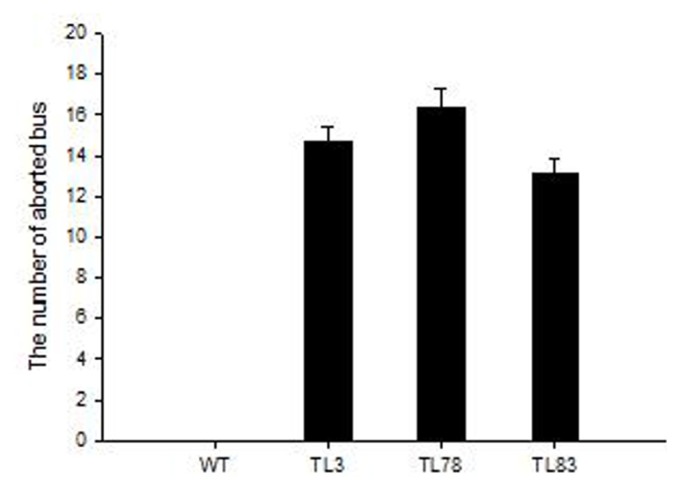
The number of aborted flower buds on the primary inflorescence of *Arabidopsis* plants subjected to heat stress. WT, Col-0 of *Arabidopsis thaliana*; TL3, TL78, TL83 are homozygous transgenic lines. Error bars represent the mean ± SD of three independent biological replicates.

**Figure 8:**
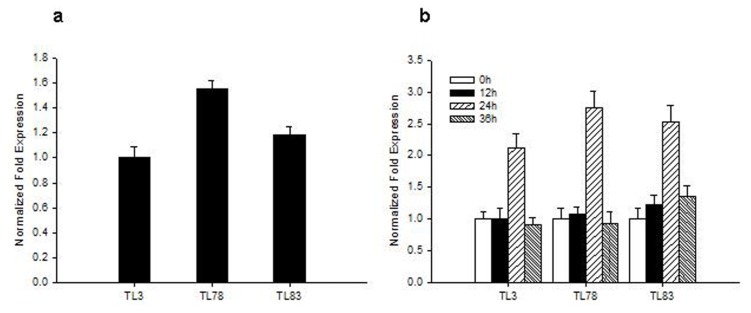
The expression of *RsVPE1* gene in transgenic *Arabidopsis* flower buds. (**a**) *RsVPE1* expression in transgenic plants under normal condition. The transcript level of *RsVPE1* in TL3 was used as the calibrator; (**b**) *RsVPE1* expression in transgenic plants subjected to heat stress. The transcript levels of *RsVPE1* in three transgenic lines at 0 h heat stress were used as the calibrators, respectively. TL3, TL78, TL83 are homozygous transgenic lines. Error bars represent the mean ± SD of three independent biological replicates.

Col-0 had been used as a model of heat-tolerance research in *Arabidopsis* accessions and reported that aborted flower buds were not observed in plants exposed to 32 °C or 34 °C, regardless of the duration of the exposure [8]. Therefore, transgenic plants were subjected to 32 ± 0.5 °C for 36 h in our study. In accordance with the observations reported by Warner *et al.* [8], aborted flower buds were not found in Col-0. However, significant phenotypic changes, in the form of aborted flower buds, were observed in transgenic *Arabidopsis* plants in response to heat stress. There were 13.1 to 16.4 aborted flower buds in three transgenic lines. FBA began to display on the third day after heat stress. All aborted flower buds appeared on the eighth day after heat stress (Figures 6 and 7). Based on the flower bud ages and positions, we could speculate that FBA occurred in the flower buds of stage 9 to 12. Although the expression of *RsVPE1* from the constitutive 35S-promoter should not be markedly affected by environmental conditions, *RsVPE1* expression levels of three transgenic lines (TL3, TL78 and TL83) reached maximums at 24 h heat stress and were shown 2.1-fold, 2.8-fold and 2.5-fold increase compared with their expression levels at 0 h, respectively (Figure 8b). In addition, *RsVPE1* expressed in transgenic lines under normal condition (Figure 8a), but aborted flower buds were not observed (Figure 7). These results suggested that *RsVPE1* gene and heat stress were important to the occurrences of FBA in transgenic *Arabidopsis* plants. Perhaps, heat stress promoted the *RsVPE1* expression to reach a particular threshold for FBA, or the heat stress induced other metabolisms, which assisted *RsVPE1* to cause FBA in transgenic *Arabidopsis* plants. Furthermore, studies have provided evidence that there were typical characteristics of PCD during FBA in radish and vegetative type VPEs acted as a key factor initiating in PCD [17,18]. As Figure 4 shown, RsVPE1 is a member of the vegetative type of VPEs. It could be assumed that *RsVPE1* expression was up-regulated to a level that is sufficient to promote programmed cell death (PCD), leading to FBA.

## 3. Experimental Section

### 3.1. Plant Materials and Growth Conditions

Radish FBA material L9 was used in this study (Figure 1) (provided by the Chinese Cabbage Laboratory of the Northwest A&F University, Yangling, Shaanxi, China). Radish plants were planted in the greenhouse in spring.

### 3.2. Cloning and Sequence Analysis of *RsVPE1* Gene

Genomic DNA was isolated from leaves using a modified CTAB protocol [19]. Total RNA was isolated from radish floral buds with TRIZOL™ according to the manufacturer’s instructions (Invitrogen, Carlsbad, CA, USA). The complete 5′ and 3′ regions of the putative *RsVPE1* gene were isolated by RACE. Approximately 1 μg of total RNA was used initially for first-strand synthesis using the SMART™ RACE cDNA Amplification Kit according to the manufacturer’s instructions (Taraka, Dalian, China). The gene-specific primers 5′GSP (5′-GATGAGTCCTGACCGACCCAGAACCTAC A-3′) and 3′GSP (5′-CCCTCCACCTGAGTATGAAACTTGCTTAG-3′) were used for 5′-RACE and 3′-RACE, respectively. The universal primers for 5′-RACE and 3′-RACE were provided in the kit. The full-length cDNA and gDNA of the *RsVPE1* gene was obtained by PCR amplification using forward (5′-ATGCCACCTGTCCCCGTCGCCGTT-3′) and reverse (5′-TTAAGCACTGAATCCACGGTGA-3′) primers. The PCR fragments were cloned into the pMD18-T vector (Takara,) and sequenced by Sangon Biotech Co., Ltd. (Shanghai, China).

NCBI bioinformatics tools were used to compare and analyze the nucleotide and protein sequence data. Multi-sequence alignment was conducted using ClustalX version 1.83 with default parameters. The phylogenic tree was built using the neighbor-joining (NJ) method using MEGA software (version 5.0) [20].

### 3.3. Tissue-Specific Expression Patterns of the *RsVPE1* Gene in Radish

To analyze tissue-specific expression of the *RsVPE1* gene in radish plants, leaves, flower stalks, flower buds, young siliques, sepals, petals, stamens and pistils of normal and aborted flower buds were collected from radish FBA material L9. Total RNA was extracted from samples using Trizol™ according to the manufacturer’s instructions (Invitrogen, Carlsbad, CA, USA). First-strand cDNA was obtained using the PrimeScript™ RT reagent Kit (Takara, Dalian, China). The EF-1-alpha gene (GO479260) (forward primer 5′-ATACCAGGCTTGAGCATACCG-3′ and reverse primer 5′-GCCAAAGAGGCCATCAGACAA-3′) was used as a loading control. The specific primer sequences used for expression analysis were as follows: 5′-TCCTGACCGACCCAGAACCTA-3′ and 5′-ATACAGTGTTGCCTGGATGGAAG-3′. Real-time PCR reactions were repeated three times in a 20 μL reaction containing 10.0 μL SYBR Premix Ex Taq, 0.8 μL forward primer (10 μM), 0.8 μL reverse primer (10 μM), 2 μL diluted cDNA and sterile ddH2O with a Bio-Rad iQ5 instrument (Foster City, CA, USA). Thermal cycling parameters were as follows: 3 min at 95 °C and 40 cycles of 20 s at 95 °C, 30 s at 58 °C and 30 s at 72 °C. The relative mRNA levels of *RsVPE1* were normalized to those of EF-1-alpha using the 2^−ΔΔ^*^C^*^t^ method [21].

### 3.4. Genetic Transformation of *RsVPE1* into *Arabidopsis*

The open reading frame (ORF) of *RsVPE1* was cloned into the pCAMBIA2301-35S-Nos vector (Cambia, Canberra, Australia). The Col-0 of *Arabidopsis thaliana* was used in this study. The plants were grown under 14-h light/10-h dark conditions at 24 °C during the day and 20 °C at night (24/20 °C) for transformation. The constructs were transferred into Agrobacterium tumefaciens GV3101 and transformed into Col-0 using the method by Clough *et al.* [22]. The seeds of the T_0_ generation were harvested and placed on MS [23] agar medium (0.8%) containing 50 μg·mL^−1^ kanamycin monosulfate (Km) after surface sterilized using a liquid method. Then the surviving transformants (T_1_) were confirmed by PCR to amplify *RsVPE1* gene and the *NPTII* gene. The confirmed transgenic plants were harvested individually. The T_2_ seeds were placed on MS agar medium (0.8%) containing 50 μg·mL^−1^ Km and the transgenic lines with a 3:1 segregation ratio (resistant:sensitive) were selected to produce T_3_ seeds. The T_3_ lines displaying 100% Km resistance were considered to be homozygous for the transgenic gene and used for further experiments. The Col-0 was used as a control. All seeds of Col-0 and transgenic plants were collected at the same stage.

### 3.5. Heat Stress on Transgenic Plants

The seeds of transgenic plants and Col-0 were fully germinated on MS agar medium for 10 days. The seedlings were then transferred from the medium to artificial soil containing peat, vermiculite and pearlites. To identify the effect of heat stress, transgenic and Col-0 plants were grown in the 24/20 °C chamber until the first flower opened. Ten plants of each type remained in the 24/20 °C chamber, while another ten plants of each type were transferred to an illumination growth chamber maintained at a constant air temperature (32 ± 0.5 °C) and relative humidity (82% ± 10%) under the same photoperiod and irradiance for 36 h. The plants were then moved back to the 24/20 °C chamber. The number of flower buds that failed to open (aborted floral buds) was determined on the eighth day after treated with 32 ± 0.5 °C for 36 h.

### 3.6. Analysis of *RsVPE1* Gene Expression in Transgenic *Arabidopsis* Plants

To evaluate the expression levels of *RsVPE1* gene in transgenic *Arabidopsis* plants under normal condition, flower buds were collected from Col-0 and three independent transgenic lines, then frozen in liquid nitrogen and stored at −80 °C for future use. In addition, to analyze the effects of heat stress on *RsVPE1* expression levels, total RNA was extracted from *Arabidopsis* flower buds of different time points during heat stress using Trizol™ according to the manufacturer’s instructions (Invitrogen, Carlsbad, CA, USA). First-strand cDNA was obtained using the PrimeScript™ RT reagent Kit (Takara, Dalian, China). Real-time PCR was conducted using the first-strand cDNA templates to detect the expression level of *RsVPE1* in *Arabidopsis* flower buds. The *Arabidopsis* AtACTIN gene (forward primer 5′-ACGTCGGAGACGAGGCACAG-3′ and reverse primer 5′-AGCGGCGCTTCG GTTAGCAAT-3′) was used as a loading control. The specific primer sequences used for expression analysis were as follows: 5′-TTCTTCCGCCCTCCCAACAAC-3′ and 5′-ATCCGCCTGATGCCTGT AGTTC-3′. Real-time PCR reactions were repeated three times in a 20 μL reaction containing 10.0 μL SYBR Premix Ex Taq, 0.8 μL forward primer (10 μM), 0.8 μL reverse primer (10 μM), 2 μL diluted cDNA and sterile ddH2O with a Bio-Rad iQ5 instrument (Foster City, CA, USA). Thermal cycling parameters were as follows: 3 min at 95 °C and 40 cycles of 20 s at 95 °C, 30 s at 58 °C and 30 s at 72 °C. The relative expression levels of *RsVPE1* were normalized to those of AtACTIN using the 2^−ΔΔ^*^C^*^t^ method [21].

## 4. Conclusions

In conclusion, a *VPE* gene, named *RsVPE1*, was isolated from flower buds of radish. *RsVPE1* transcript levels varied in different tissues of radish plants. The highest *RsVPE1* expression was detected in aborted flower buds, which indicates that *RsVPE1* plays an important role in FBA. Although *RsVPE1* was expressed in three independent homozygous transgenic *Arabidopsis* lines, no obvious morphological or developmental abnormalities were observed in transgenic plants when these plants were grown in artificial soil at normal temperatures. However, aborted flower buds were observed in transgenic *Arabidopsis* plants in response to heat stress. In addition, *RsVPE1* expression levels reached maximums at 24 h heat stress and were shown 2.1-fold to 2.8-fold increase compared with their expression levels at 0 h, respectively. These results implied that *RsVPE1* gene and heat stress were important to the occurrences of FBA in transgenic *Arabidopsis* plants. The data also demonstrated that *RsVPE1* played an important role in flower development of radish and transgenic *Arabidopsis* plants. When the expression level of *RsVPE1* reaches a particular threshold, the development of flower buds is hindered. It could be assumed that *RsVPE1* expression was up-regulated to a level that is sufficient to promote programmed cell death (PCD), leading to FBA.
